# Endocrine manifestations of lung adenocarcinoma with epidermal growth factor receptor mutation mimicking tuberculosis: A case report and literature review

**DOI:** 10.5339/qmj.2025.64

**Published:** 2025-06-09

**Authors:** Lara Arafsha, Shaza A. Samargandy, Anas S. Alyazidi

**Affiliations:** 1 Department of Internal Medicine, Faculty of Medicine, King Abdulaziz University, Jeddah, Saudi Arabia; 2 Faculty of Medicine, King Abdulaziz University, Jeddah, Saudi Arabia *Email: alyazidi.anas@gmail.com

**Keywords:** Lung adenocarcinoma, metastatic lung adenocarcinoma, pituitary metastases, metastasis, miliary tuberculosis

## Abstract

**Background:**

Lung cancer is a leading cause of cancer-related mortality globally, often presenting with diverse and challenging manifestations. This case report discusses an unusual presentation of epidermal growth factor receptor (EGFR)-mutated non-small-cell lung cancer (NSCLC) initially mimicking tuberculosis (TB), complicated by pituitary involvement.

**Case Presentation:**

A 30-year-old female presented with respiratory symptoms and systemic complaints, initially suggestive of miliary TB. Further investigations revealed metastatic lung adenocarcinoma with pituitary metastasis, causing diabetes insipidus, hyperprolactinemia, adrenal insufficiency, and hypothyroidism. Treatment with targeted therapy involving osimertinib resulted in clinical improvement.

**Conclusion:**

This case underscores the diagnostic challenges posed by atypical presentations of lung cancer, which can masquerade as infectious diseases like TB. The presence of pituitary metastasis further complicates the clinical picture, emphasizing the importance of considering rare metastatic sites in the differential diagnosis of lung adenocarcinoma. Timely recognition and appropriate management are crucial for optimizing outcomes in such complex cases, highlighting the need for a multidisciplinary approach in oncological and endocrine care.

## Introduction

Globally, lung cancer is the second most commonly diagnosed cancer and the leading cause of cancer-related mortality.^1^ The disease can sometimes present atypically, complicating the diagnostic process.^1^ For example, diffuse pneumonic-type primary lung adenocarcinoma can resemble inflammatory or infectious lung diseases, making accurate diagnosis challenging.^2^ A miliary pattern on chest imaging can be indicative of various conditions, both benign and malignant. Benign causes include fungal and bacterial infections and occupational lung disorders.^3^ More severe conditions that can present with a miliary pattern include metastatic lung cancers, particularly adenocarcinoma, and miliary tuberculosis (TB), which can present in a similar way.^4^ Miliary TB, in particular, can manifest with nonspecific symptoms such as fever, weight loss, sweats, and anorexia, which can mimic the presentation of primary and secondary lung cancers.^4^ Lung adenocarcinoma can lead to several complications, including rare but severe endocrine metastases. According to one study, metastases to the pituitary gland, although rare, have a poor prognosis.^5^ The most frequent presenting symptom of pituitary metastases is diabetes insipidus (DI), but patients may also experience hyperprolactinemia, adrenal insufficiency, and thyroid insufficiency.^5^ The complexity of these presentations poses significant challenges to healthcare providers, especially in rapidly progressing conditions. This case report highlights the difficulties in recognizing and treating atypical presentations of lung cancer.

## Case Presentation

Following the CARE (CAse REport) guidelines for reporting case reports,^6^ we present a case in 2024 involving a 30-year-old Yemeni female who arrived at the emergency department (ED) of King Abdulaziz University Hospital in Jeddah, a public healthcare facility. The patient reported an intermittent dry cough for 2 weeks that had increased in frequency prior to presenting to the hospital, associated with pleuritic chest pain predominantly on the left side, and nonprogressive shortness of breath. Her medical history was unremarkable, with no known chronic illnesses and no history of smoking. Prior to her visit to our ED, she had been evaluated at a secondary hospital where contrast-enhanced chest CT revealed pulmonary miliary nodules and a right lobe mass, prompting a referral to a tertiary center. She was discharged with oral antibiotics, which provided slight symptom relief. Due to the patient’s symptoms and the suspicion of miliary nodules suggestive of pulmonary tuberculosis, the patient was initially isolated in the isolation unit. At our hospital, she reported new symptoms: fatigue, night sweats, rigors, a 10-kilogram (kg) weight loss over 2 months, and persistent blurry vision in her left eye. She also had left hip pain radiating to the foot and exacerbated by movement, along with excessive thirst (5L/day) and polyuria. Additionally, she experienced galactorrhea and amenorrhea for 5 months following postpartum irregular menstruation and lactation, which ceased 1 month prior.

During the general physical examination, the patient was alert and oriented. Her vital signs revealed a blood pressure of 100/72mmHg, a heart rate within the normal range, and an oxygen saturation of 97% on a three-liter nasal cannula. There were no signs of distress. Cardiovascular examination was normal, with regular heart sounds and palpable peripheral pulses. Lung examination revealed decreased bilateral air entry at the bases with scattered crepitations. The abdomen was soft, nontender, nondistended, and without palpable masses. There were no signs of lower limb edema or deep vein thrombosis. Fundus examination was performed due to the patient’s visual complaints, revealing flat, hypopigmented retinal lesions in the right eye and an elevated dome-shaped lesion with hyperpigmented focal spots in the left eye.

**Figure 1:**
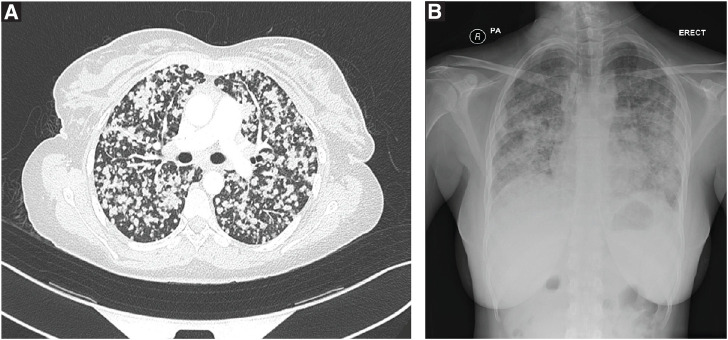
(A) CT scan of the chest showing many bilateral pulmonary nodules with a miliary-type appearance throughout both lung fields. (B) Chest X-ray with multiple nodular opacities scattered throughout both lung fields.

**Figure 2:**
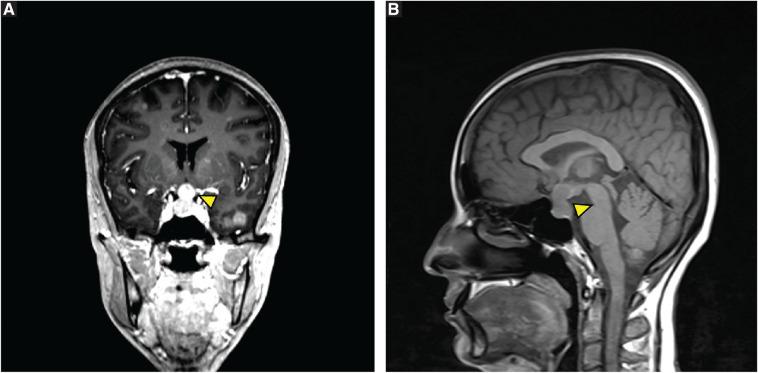
Brain MRI (A) Pituitary enhanced by contrast. (B) Pituitary enlargement. Arrowheads indicate the site of the lesion.

Radiologically, the patient initially presented with a bilateral distribution of multiple small nodules, which were scattered throughout both lungs, a pattern typically seen in miliary TB (Figure 1). Magnetic resonance imaging (MRI) of the brain and orbits showed multiple supratentorial and infratentorial juxtacortical and deep white matter lesions, as well as a well-defined bilobed sellar and suprasellar lesion indicative of a metastatic process (Figure 2). A plaque-like lesion in the superior posterolateral aspect of the left orbital globe was consistent with choroidal metastasis.

Further workup included bronchial wash for malignancies and acid-fast bacilli (AFB) stain and culture, both of which were negative. The purified protein derivative (PPD) test was also negative. A computed tomography (CT)-guided biopsy of a sacral lesion revealed histopathological findings consistent with metastatic adenocarcinoma of lung origin. Notably, the epidermal growth factor receptor (EGFR) mutation was positive, identifying the cancer as non-small-cell lung cancer (NSCLC). Given the sellar and suprasellar mass, clinical suspicion was high for diabetes insipidus and possible hypopituitarism. Laboratory results included follicle-stimulating hormone (FSH) at 0.53 mIU/mL (normal: 3.03–8.08 mIU/mL), luteinizing hormone (LH) <0.12 mIU/L, serum sodium 136 mmol/L, serum potassium 4.4 mmol/L, prolactin 2975.42 mIU/L, cortisol <27.6 μg/dL, free thyroxine (FT4) <5.41 pmol/L, free triiodothyronine (FT3) 2.35 pmol/L, and thyroid-stimulating hormone (TSH) 2.6833 mIU/L. An overnight fast from all liquids was conducted, and urine chemistry the next morning showed urine sodium 50.0 mmol/L, urine osmolality 124.9 mOsmol/kg, and serum osmolality 296mOsmol/kg. In conjunction with the suprasellar mass, these findings confirmed central diabetes insipidus.

The patient was started on hydrocortisone therapy, followed by thyroxine therapy the subsequent week. Desmopressin therapy was initiated, leading to improvement in polyuria and polydipsia, with stable sodium levels. Tyrosine kinase inhibitor therapy was started, and a diagnosis of hypopituitarism secondary to metastatic lesions was made, encompassing partial central diabetes insipidus, adrenal insufficiency, and central hypothyroidism. The hyperprolactinemia was attributed to the stalk effect. The patient received osimertinib targeting the EGFR mutation, resulting in clinical improvement, and was discharged 12 weeks after her initial presentation with significant improvement. The patient was lost to follow-up after relocating to her home country to undergo the recommended treatment. To ensure continuity of care, she was provided with her complete medical records.

## Discussion

Lung cancer is the second most commonly diagnosed cancer globally,^1^ accounting for nearly one-tenth of all cancer cases^7^ and leading to nearly 18% of all cancer-related deaths, making it the primary cause of cancer mortality.^1,8^ The presentation of the disease can be variable, with several atypical presentations reported in the literature.^2^ Among such presentations are patients presenting with adenocarcinomas mimicking TB (Table 1). Furthermore, the World Health Organization (WHO) has reported that tuberculosis cases have surged, making it the leading infectious killer disease, surpassing COVID-19 in mortality.^9^ These cases have been described repeatedly in the literature, leading to prolonged diagnostic challenges and possibly unnecessary or inaccurate management plans.^4,10–13^

In our case, the patient was initially suspected to have miliary TB due to the regional prevalence, which have seen a surge of TB cases, and her signs and symptoms were suggestive of pulmonary TB, contributing to her primary differential diagnosis at the time. The challenges arose because of the overlapping features similar to those noted in diagnosing miliary TB, where vague symptoms and extrapulmonary organ involvement complicate timely diagnosis and treatment, which is crucial for patient survival.^14^ In certain instances, TB can mimic lung cancer, posing diagnostic difficulties in clinical settings.^15^ As reported similarly in cases resembling ours in regions with high TB prevalence, such cases must be considered, especially in patients with nonspecific symptoms and miliary shadows on chest imaging, necessitating further evaluations to confirm the diagnosis.^4^

Furthermore, our patient’s young age posed an additional challenge, similar to cases noted previously, advising a high index of suspicion in relatively young adult smokers with unusual presentations.^10^ Diagnostic complexity was also heightened by CT findings suggestive of miliary TB, mirroring results from other similar complaints.^11^ Notably, our patient exhibited pituitary symptoms, prompting concern due to worsened symptoms alongside an enlarging pituitary mass and widespread metastases, which are indicative of pituitary metastasis. This scenario aligns with findings in another case where a bone biopsy revealed poorly differentiated lung adenocarcinoma, paralleling our sacral cone biopsy.^16^

Pituitary metastasis is rare, representing <1% of intracranial metastases.^12^ Treatment of adrenal insufficiency should precede the treatment of hypothyroidism to avoid the risk of adrenal insufficiency crisis. Additionally, steroid treatment for adrenal insufficiency can unmask and exacerbate diabetes insipidus, as evidenced in this patient, where the requirement for desmopressin increased after hydrocortisone therapy.^17^

Additionally, osimertinib was advised to follow the same guidelines.^18^ This intervention led to a notable improvement in the patient’s condition. Consequently, the patient was discharged with better-managed symptoms related to malignancy and endocrine issues. Similar to a case reported in the literature with an EGFR mutation,^13^ our case underscores the importance of considering pituitary metastasis in the differential diagnosis of middle-aged patients with lung adenocarcinoma, which can mimic miliary TB at presentation.

## Conclusion

In this study, we emphasize the crucial significance of considering lung adenocarcinoma with pituitary metastasis as a potential diagnosis when patients manifest symptoms mimicking miliary TB and features of endocrine dysfunction and/or diabetes insipidus. This case highlights the significant challenges in diagnosing and managing atypical presentations of lung cancer, particularly when they mimic common infectious diseases such as miliary TB. Our approach underscores the importance of timely and accurate diagnosis in improving patient care and managing conditions related to malignancy and endocrine disorders. The patient was successfully managed with hydrocortisone, desmopressin, levothyroxine, and osimertinib therapy. Following this treatment, the patient showed significant improvement in both malignancy-related symptoms and endocrine issues, resulting in a favorable outcome upon discharge.

## Ethical approval

Written informed consent was obtained to publish the details of the reported case from the patient’s legal parents after describing the nature of the published information, its uses, and the research objectives. Our institution does not require ethical approval to report individual cases. All identifying information was masked to ensure patients’ privacy at all stages of this study. The study adhered to the Declaration of Helsinki.

## Conflicts of interest

There are no conflicts of interest to disclose.

## Figures and Tables

**Table 1.** Cases review of cancer mimicking miliary tuberculosis with endocrine manifestations in the literature.

**Author**	**Date**	**Gender**	**Age (in years)**	**Immunohistochemical/genetic analysis**	**Type of lung cancer**	**Pituitary metastasis**	**Mimicking**
Arafsha et al.	2024	Female	30	Positive EGFR mutation	Adenocarcinoma	+	Miliary TB
Seifi et al.^4^	2023	Male	25	NM	Adenocarcinoma	NM	Miliary TB
Asim et al.^13^	2023	Male	54	Positive EGFR mutation—Exon 19 deletion	Lung adenocarcinoma	+	NM
Hamada et al.^12^	2021	Female	68	NM	Nonmucinous pulmonary micropapillary adenocarcinoma	NM	Pulmonary TB
Khan et al.^11^	2020	Male	35	Positive napsin A and ALK 5A4	Pulmonary mucinous adenocarcinoma	NM	Miliary TB
Zia et al.^10^	2020	Male	36	Strongly positive for PD-L1	Lung adenocarcinoma	NM	Miliary TB

EGFR: epidermal growth factor receptor, TB: tuberculosis, NM: not mentioned, ALK 5A4: anaplastic lymphoma kinase 5A4, PD-L1: programmed cell death ligand 1.
